# Supplementary material for: County-level factors affecting Latino HIV disparities in the United States
Source: PLoS One. 2020 Aug 12;15(8):e0237269. doi: 10.1371/journal.pone.0237269 (PMC7423131; doi:10.1371/journal.pone.0237269)
Supplement: S1 Table — (PDF) [file pone.0237269.s001.pdf]

S1 Table. Description and Data Sources for County-Level Factors

Category	Variable	Description
**HIV Characteristics**	County Prevalence Rate[a]	Prevalence rate of HIV per 100,000 in each county
	Male Prevalence Rate[a]	Prevalence rate of HIV per 100,000, males only
	Female Prevalence Rate[a]	Prevalence rate of HIV per 100,000, females only
	% MSM[a]	% of HIV cases attributed to men who have sex with men
	% IDU[a]	% of HIV cases attributed to injection drug use
	% Heterosexual[a]	% of HIV cases attributed to heterosexual sex
	New Diagnosis Rate[d]	Prevalence rate of new diagnoses of HIV, per 100,000, in each county in 2016
	IDU Prevalence Rate[a]	Prevalence rate of HIV cases, per 100,000 population
	MSM Prevalence Rate[a]	Prevalence rate HIV cases, per 100,000 attributed to MSM
**Socioeconomic**	[a]Median Household Income[a]	Median household income
	GINI Index[a]	Measure of statistical dispersion to represent income distribution
	% High School Education[a]	% of population with a high school degree or equivalent
	% Unemployed[b]	% of county population that is unemployed
	Income Inequality Ratio[b]	Ratio of household income at 80th percentile to income at 20th percentile
	Non-White/White Segregation Index[b]	The degree to which non-white and white live separately from one another
	Black/White Segregation Index[b]	The degree to which black and white live separately from one another
	% Non-Latino White Living Below Poverty[c]	% of non-Latino white population in each county living below the poverty line
	% Living Below Poverty[c]	% of total county population living below the poverty line
**Community Environment**	% Single Parent Household[b]	% of households in each county that are single parent
	Social Association Rate[b]	# of membership associations per 10,000 population
	Violent Crime Rate[b]	# of reported violent crime offenses per 100,000 population
	% Severe Housing Problems[b]	% of households with at least 1 of 4 housing problems: overcrowding, high housing costs, lack of kitchen or lack of plumbing
	% Rural[b]	% that is not urban
**Health Behaviors**	% Excessive Drinking[b]	% of adults reporting binge or heavy drinking
	Chlamydia Rate[d]	# of newly diagnosed chlamydia cases per 100,000 population
	Drug Overdose Mortality Rate[b]	Drug overdose-related mrtality per 100,000 population
	Gonorrhea Rate[b]	# of newly diagnosed gonorrhea cases per 100,000 population
**lth Care**	% Uninsured[a]	% of population in under age 65 without health insurance
	Primary Care Provider Rate[b]	Ratio of population to primary care physicians

**Access to Heal**	Mental Health Provider Rate[b]	Ratio of population to mental health providers
	Preventable Hospitalization Rate[b]	# of hospital stays for ambulatory-acre sensitive conditins per 1,000 Medicare enrollees
	Healthcare Costs[b]	Amount of price adjusted Medicare re-imbursement
**Latino Characteristics**	Latino Population % Change 2000-2014[e]	% change in Latino population from 2000 to 2014
	% Mexican[e]	% of Latinos that identify as Mexican
	% Puerto Rican[e]	% of Latinos that identify as Puerto Rican
	% Latino[b]	% Latino population in each county
	% Not English Proficient[b]	% of population that is not proficient in English
	% Latino Living Below Poverty[c]	% of Latino population in each county living below the poverty line
**Latino/NL-White Ratios**	Poverty Ratio[c]	% Latino Below Poverty / % Non-Latino White below poverty
	Population Ratio[f]	% Latino population / % Non-Latino White population
	Latino/Non-Latino White Income Ratio[b]	Latino Income / Non-Latino White Income

Data Sources:

[a] AIDSVu. Emory University, Rollins School of Public Health. Atlanta, GA. Available at: https://aidsvu.org/resources/#/2016. Accessed November 3, 2019. Dataset: AIDSVu_National_Prev_2016.xlsx.

[b] University of Wisconsin Population Health Institute. County Health Rankings and Roadmaps. Madison (WI): University of Wisconsin Population Health Institute. [cited 2019 November 3]. Available from: https://www.countyhealthrankings.org/explore-health-rankings/rankings-data-documentation/national-data-documentation-2010-2018. Dataset: 2018 County Health Rankings Data - v2.xlsx

[c] United States Census Bureau. American Fact Finder [Internet]. United States Census Bureau; Table S1701: poverty status in the past 12 months, 2013---2017 American Community Survey 5-year estimates. Available at: https://factfinder.census.gov/faces/tableservices/jsf/pages/productview.xhtml?pid=ACS_17_5YR_S1701. Accessed November 3, 2019.

[d] Centers for Disease Control and Prevention. NCHHSTP AtlasPlus. Updated 2019. https://www.cdc.gov/nchhstp/atlas/index.htm. Accessed [illegible]

[e] 1) United States Census Bureau. American Fact Finder [Internet]. United States Census Bureau; Table B01003: American Community Survey 5-Year Estimates, 2011-2015. Available at: https://factfinder.census.gov/faces/tableservices/jsf/pages/productview.xhtml?pid=ACS_15_SPT_B01003. Accessed November 3, 2019.; 2) United States Census Bureau. American Fact Finder [Internet]. United States Census Bureau; 2000 population estimates (http://www.census.gov/popest/). Accessed: 11/02/2019.

[f] Population Ratio calculated using AIDSVu data
