# Supplementary material for: County-level factors affecting Latino HIV disparities in the United States
Source: PLoS One. 2020 Aug 12;15(8):e0237269. doi: 10.1371/journal.pone.0237269 (PMC7423131; doi:10.1371/journal.pone.0237269)
Supplement: S1 Text — (PDF) [file pone.0237269.s002.pdf]

S2 Technical Appendix

I. **Correcting for Measurement Error in Correlations**.

To correct for measurement error in the correlation between the two prevalence estimates on a logarithmic scale, we transform the variances using the Delta Method. Specifically, since the measurement error variance for each county's Latino rate is

$$\text{Var}\ (Rate_{Latino}) \sim Rate_{Latino}\ /\ N_{Latino},$$

then

$$\text{Var}\ (log\ Rate_{Latino}) \sim \text{Var}\ (Rate_{Latino})\ /\ (Rate_{Latino})^2 = 1\ /\ (N_{Latino}\ Rate_{Latino})$$

and similarly for the NL White variance.

We used Mplus to correct for these known variances in the measures using the following code.

```
VARIABLE: NAMES ARE
  logL selogL logNL selogNL # log and standard errors for log PRRs for Latino and White NL
    USEVARIABLES ARE logL  logNL ;
 ANALYSIS:  type = random;
  MODEL:
     betaL | logL on selogL;  betaL @1;  [betaL @0]; # logL has measurement std error selogL
     betaNL | logNL on selogNL; betaNL @1; [betaNL @0]; # similar for logNL
     betaNL on betaL @ 0; # measurement errors are independent
```

logL with logNL; # correlation adjusted for measurement error.

II. Calculating Unexplained Deviance in the Population Risk Ratio

Unexplained deviance in the population risk ratio for Latino versus NL White prevalence rates of HIV is defined in terms of two components derived from that model: the coefficient of disparity on a logarithmic scale, and the residual variation in this log disparity across counties that is not explained by county level covariates and their interactions with race/ethnicity (i.e., the random effect variance). We include the coefficient of disparity as unexplained deviance as this would be zero (on a log scale) if the other terms in the model explained the mean (log) disparity entirely. In each of our bivariate models, we include two county-level random effects, one for Latinos and NL Whites, and allow them to be correlated with one another so that we could model each racial/ethnic group's prevalence rate of diagnosed HIV infection separately as a function of single or multiple covariates, as well as use this same model to account for disparity itself. That is, because

$$\log(PRR) = \log(Rate_{Latino}) - \log(Rate_{NL\ white})$$

our two correlated random effects, one for Latino and one for NL Whites can appropriately account for county-level variability in the disparity since

$$\begin{aligned}\text{Var}\ (\ \log(PRR)) = Var\ \ \log(Rate_{Latino}) - \\ 2\ Cov\ (\log(Rate_{Latino})\ , \log(Rate_{NL\ white})) + \\ Var\ (\log(Rate_{NL\ white})).\end{aligned}$$

The three terms on the right side of this equation are all estimated in these two-level mixed effects models.

III. Bivariate Modeling for Examining Homogeneous and Moderator Effects

To examine the homogeneous effect of a covariate on both Latino and NL White prevalence of diagnosed HIV infection, we include this covariate term as having the same, or main effect, across these two ethnic groups.

To examine the moderating effect of how the covariate's effect varies by Latino and NL White prevalence of diagnosed HIV infection, we include the corresponding interaction term in each of the GLMMs we examine.

In particular, our bivariate modeling uses as an outcome Y_{ij}, the number of persons with diagnosed HIV infections in county i for race/ethnicity j, where i indexes the county and $j = 1, 0$, indexing Latino and White NL rates. We use the following coding to produce parameter estimates of interest. Let NL_{ij} denote a (negative) binary indicator for NL White prevalence of diagnosed HIV infection within county, where $NL = 0$ for Latinos and $NL = -1$ for NL Whites. Let X_i denote a county-level covariate, which is the same for both ethnicities, and pop_{ij} denote the ethnic specific population estimate. The full model for estimating race/ethnicity specific prevalence of diagnosed HIV infection based on a vector of covariates **X** is given by the following mixed-effects Poisson regression model to predict Y's expected value with an offset,

$$\log(E\, Y_{ij}) = \beta_0 + \beta_1 NL_{ij} + \boldsymbol{\beta'}_2 \boldsymbol{X_i} + \boldsymbol{\beta_3}' \boldsymbol{X_i} NL_{ij} + b_{i0} + b_{i1} NL_{ij} + \log(pop_{ij}) \quad \text{(Model 1)}$$

With this coding, β_0 corresponds to the log of the Latino prevalence of diagnosed HIV rate, β_1 is the log disparity rate of Latino vs. NL White adjusted for other terms in the model; $\boldsymbol{\beta}_2$ is the vector of the main effects of **X**, and $\boldsymbol{\beta_3}$ accounts for the moderating effect of **X** on the log of the race/ethnicity-specific observed counts in county i. The coefficients b_{i0} and b_{i1} are county-specific random effects for Latinos and the disparity between Latinos and NL Whites. These follow a bivariate normal distribution with mean 0 and 2-dimensional variance-covariance

matrix Σ. With this coding, the random effect for a count's NL White rate is given by $b_{i0} + b_{i1}$. Examining the coefficient β_1 as well as the variance of $z_i = b_{i0} + b_{i1}$ for models with multiple covariates shows how much these covariates explain the deviation in disparities from zero. Allowing for a correlation between these two coefficients (b_{i0}, b_{i1}) permits an individual county's Latino and NL White prevalence rates of diagnosed HIV infection on average to be higher or lower than that predicted by the model.

To relate this to covariate adjustment for homogeneous and moderation effects, we first consider the case where there the interaction parameters $\boldsymbol{\beta_3} = \mathbf{0}$. The effect β_1 represents the effect of race/ethnicity on the outcome once we adjust for or control for all covariates **X,** including any interaction terms. When the interaction parameters $\boldsymbol{\beta_3}$ are zero, we refer to this type of model as a covariate main effect adjusted analysis.

We note that Model 1 also expresses a model that accounts for how an individual county's population risk ratio (PRR) or Latino disparity is affected by a county-level covariates $\boldsymbol{X_i}$.

$$\log(PRR_i) = \log(E\ Y_{i1} \,/\, pop_{i1}) - log(E\ Y_{i0} \,/\, pop_{i0})) = \beta_1 + \boldsymbol{\beta}_3'\boldsymbol{X_i} + z_i$$

where again $z_i = b_{i0} + b_{i1}$ is the random effect for the log disparity. since the terms in the parentheses are both error terms with zero means, the effect of the baseline variables $\boldsymbol{X_i}$ on the PRR changes the mean depending on coefficients $\boldsymbol{\beta}_3'$ and changes the residual variance as well.

IV. Comparing How Much Disparity is Explained and Remains Unexplained by Covariate Homogeneity and Moderator Terms

Formally, we define unexplained disparity in a model as the average squared deviation from 0 of the log PRR values after adjustment for terms involving the covariates. Here we

present our means of comparing how much unique explanatory power rests with the covariates, first as multiple main effects and then as moderators. We begin with the unadjusted log PRR point estimate, computed without any covariates in the following simple model.

$\log(E\ Y_{ij}) = \beta_0 + \beta_1(Model\ 0)\ NL_{ij} + b_{0i} + b_{1i}NL_{ij} + \log(pop_{ij})$ (Model 0)

Averaging over all the counties, the mean log PRR is, referring to Model 0 above,

Ave log PRR_i = $Ave\ (\log(E\ Y_{i1}\ /\ pop_{i1}) - log(E\ Y_{i0}\ /\ pop_{i0}))\ =\ \beta_1\ (Model\ 0)$ + Ave $(b_{0i} + b_{1i})$

The total **unexplained disparity or deviation from 0,** is then

Unexplained Deviation = ${\beta_1}^2 + Var(\log PRR) = {\beta_1}^2 + Var(b_{1i})$

With homogeneous covariate main effects, but no moderation through covariates **X** = (X_1 , … , X_p), Model 1 is changed to

$\log(E\ Y_{ij}) = \beta_0 + \beta_1\ (Model\ 2)NL_{ij} + \boldsymbol{\beta_2}'\boldsymbol{X_i} + b_{0i} + b_{1i}NL_{ij} + \log(pop_{ij})$ (Model 2)

so the unexplained deviation involves the same parameter values under a different model:

Unexplained Deviation for Model 2 = ${\beta_1}^2\ (Model\ 2) + Var(b_{1i}\ Model\ 2)$

The **percentage of deviation explained** by Model 2 relative to that of Model 1, which is presented in Table 3, is then

Percent Explained Deviation = 100 (1 - Unexplained Deviation for Model 2 / Unexplained Deviation for Model 1)

With moderation included (Model 1), we have a new term involving the mean of all the covariates,

Ave log PRR_i = $\beta_1(Model\ 1) +\ \boldsymbol{\beta}'_3\ (Model\ 1)\overline{\boldsymbol{X}}$.

Thus by transforming the original covariates so they are centered at the mean so that $\boldsymbol{X} = \boldsymbol{0}$ and the last term drops out, the unexplained disparity is again ${\beta_1}^2(Model\ 1) + Var\ (b_{1i}\ \ Model\ 1)$. These values are provided in Table 3 in the text.

V. Examining the Potential that Confounding by Age Accounts for Variation by Race/Ethnicity

In this section we develop formulae to express how large the age-specific correlations between county level prevalence in HIV and county size would need to be in order for age to be a strong enough confounder to account for ethnic variations in prevalence. Note that race/ethnicity age-specific rates of HIV prevalence by county are not available because of suppression, so we cannot directly check for confounding by age. However, county level age by race/ethnicity denominators are available from the Census, which we define as N_{ae}^{i}, collapsed into 5 age categories a = 13-24, 25-34, 35-44, 45-54, 55+; race/ethnicity e = 1 (Latino) and e = 2 (NL White), and county i, i = 1 , …, K = 671, the largest number of counties in our analyses. Also reported are age by race/ethnicity rates of HIV prevalence at a national level, P_{ae}. Completely missing are the individual county level prevalences, P_{ae}^{i}. If age were to completely explain the differences in race/ethnicity prevalences we observe, we would require that P_{ae}^{i} be unrelated to E = e given A = a and county i. We examine the implication of this assumption below.

Define $U_{ae}^{i} = N_{ae}^{i} / N_{ae}$ as the age-race/ethnicity proportion of the ae population in the ith county. Using this notation, the national rates are just the weighted average of the county-level prevalences,

$$P_{ae} = \sum_{i=1}^{K} U_{ae}^{i} * P_{ae}^{i} \quad (1)$$

One way that age could act as a potential confounder to replace the entire effect that we observe by ethnicity, is if there are correlations between prevalence, P_{ae}^{i}, and age-specific size, $U_a{}^{i} = U_{a1}^{i} + U_{a2}^{i}$.

We assume the following linear regression model relating these two correlated factors

$$P_{ae}^{i} = \alpha_a + \beta_a U_a^{i} + b_a^{i} + \varepsilon_{ae}^{i},\ e = 1 , 2 \quad (2)$$

where β_a is the (linear) regression coefficient of county level prevalence on the proportion of the US population in that county for a specified age group, b_a^i is a random intercept accounting for county level variation with variance σ^2, and ε_{ae}^i corresponds to a binomial proportion error with

$Var(\varepsilon_{ae}^i) = P_{ae}^i(1 - P_{ae}^i) / N_{ae}^i$.

Incorporating equation (2) into (1), US population prevalences of age and race/ethnicity satisfy

$P_{ae} = \alpha_a + \beta_a (\sum_{i=1}^{K} U_{ae}^{i} * U_a^{i}) + \varepsilon_{ae}$ (3).

As the number of parameters on the right side of the equation is the same as the number of US prevalences on the left, i.e., a saturated model, estimates of the regression coefficients are explicit. In particular,

$\widehat{\beta_a} = (P_{a1} - P_{a2}) / (\sum_{i=1}^{K} U_{a1}^{i} * U_a^{i} - \sum_{i=1}^{K} U_{a2}^{i} * U_a^{i})$ (4)

We converted these regression coefficients to correlations from (2) via

$\widehat{\rho_a} = \widehat{\beta_a} \sqrt{Var(P_{ae}^i)/Var(U_a^i)}$ (5)

Inside the square root sign the $Var(U_a^i)$ are directly computable, but $Var(P_{ae}^i)$ are not because all the county level age by race/ethnicity prevalences are missing. We approximate this variance by direct calculation of the values we do have for $Var(P_e^i)$ and since $P_e^i = \sum_{a=1}^{5} w_{ae}^{i} * P_{ae}^{i}$ where the age category weights w_{ae}^i are all close to 1/5 (i.e., equal age distributions), $Var(P_{ae}^i) \sim 5\ Var(P_a^i)$ under our conditional independence model (2). These estimated correlations $\widehat{\rho_a}$ calculated from equation (5) are provided below.

Table 1. Prevalence and its Relationship with County Size for Different Age Groups

	Table A1. Prevalence and its Relationship with County Size for Different Age Groups			
Age	Population Proportion Latino	Prevalence Risk Ratio (PRR)	$\widehat{\beta_a}$	$\widehat{\rho_a}$
13-24	0.21	3.53	0.02	0.01
25-34	0.21	3.22	0.18	0.08
35-44	0.19	2.87	0.27	0.12
45-54	0.14	2.79	0.39	0.19
55+	0.08	4.97	0.21	0.10

The proportion Latino to (Latino + NLW) decreases with age while the PRRs are substantially greater than one for all age groups and highest among the oldest group. Examining the last column, our model (2), which assumes all observed differences in race/ethnicity are accounted for by age, would imply there is no relationship between HIV prevalence and county size for the youngest group ($\widehat{\rho_a} = 0.01$), but positive relationships with all other age groups; i.e., larger HIV prevalences in larger counties by age. Both the regression coefficients and correlations increase with age except for the oldest age group, with the largest correlation of 0.19 for the 45-54 age group.
