# Supplementary material for: County-level factors affecting Latino HIV disparities in the United States
Source: PLoS One. 2020 Aug 12;15(8):e0237269. doi: 10.1371/journal.pone.0237269 (PMC7423131; doi:10.1371/journal.pone.0237269)
Supplement: Attachment — Submitted filename: Response to reviewers.doc [file pone.0237269.s004.doc]

July 13, 2020

Dear Dr. Marotta:

We are resubmitting our manuscript, “County-level factors affecting Latino HIV disparities in the United States”. We are appreciate the reviewer’s comments and are pleased to respond to each point raised. The reviewers’ comments have led us to add more context and provide practical solutions to identified factors associated with disparities that we have identified in our analyses. We thank the reviewers as these have led to what we believe are improvements in the manuscript. Responses to the academic editor and reviewer(s) are described below and in our submission with tracked and untracked changes.

**Editor**

1. Please ensure that your manuscript meets PLOS ONE style requirements, including those for file naming.

Based on PLOS ONE style requirements, we have made the following changes: reformatted tables, modified font size to reflect Level 3 headings, deleted “Funding” and “Author Contribution”, reformatted figures, and renamed supporting documents files.

**Reviewer 1**

The manuscript is interesting and could be accepted after revision.

Authors should improve the discussion, better explaining the lesson learnt and possible solutions to this problem.

Specifically:

1) Reviewer comment: Authors stated: “Increasing the availability, initiation, and retention of PrEP and ART are essential strategies to eliminate HIV.”….It has to be better contextualized and explained. In which way? In fact, in the previous paragraph they stated, “In such settings, language and cultural differences could be significant barriers to HIV testing and treatment that would differentially affect Latinos across both HIV prevention and treatment cascades”…How authors think possible increase PrEP and ART? See ref: DOI: 10.1186/s12889-018-5646-8

Response: To address this and other comments by both reviewers, we restructured the discussion section completely. We addressed this comment in lines 507-523 (in track-changes version) with changes, additions, and new references that provide context and potential solutions to engagement and retention to HIV care services:

2) Reviewer comment: Authors should better explain also the following: “We conjecture that focusing primarily on changing individual level risk behavior by itself is not likely to overcome all these disparities” how much important it this for authors?

Response: In discussing this conjecture, we restructure the discussion to highlight how our findings are aligned with the literature that describes the multiple levels contributing to disparities, following a socioecological framework of health. In lines 507-523, we discuss individual and provider level barriers to access to care that can lead to disparities and identify interventions that can be adapted to address them. In lines 524-544, we present community-level factors and suggest approaches and interventions that may be tailored for Latinos to improve access and use to needed services. In lines 553-572, we discuss individual level factors associated with injection drug use. In lines 577-586, we present socioeconomic, societal level factors strongly associated with HIV and other health disparities among Latinos.

3) Reviewer comment: Authors should create a priorities list of actions to tackle this problem.

Response: We agree and this comment guided the way we restructured the Discussion section. These proposed actions and approaches address individual, community, and societal levels as described in item 2 above.

4) Reviewer comment: Social context was mentioned many time but never well discussed, maybe authors could consider these references: https://doi.org/10.1016/j.tube.2017.01.002; doi: 10.1177/00333549101250S405

Response: We appreciate this observation and it also informed restructuring of the Discussion section. We use the socioecological framework of health in which social context is a key element. To address this specifically, we added lines 524-544 that address existing research on successful outreach approaches to Latinos and new research that is needed.

**Reviewer 2**

The authors wrote a very interesting paper on the determinants of HIV disparities in a specific HIV population.
I suggest to publish it after some minor revisions

Only some suggestions to improve the manuscript:
1) Reviewer comment: reorganizes according to the editorial guidelines of the journal

Response: We thank the reviewer for pointing this out. Changes have been made and are described in item #1 in response to Editor comment.

2) Reviewer comment: Introduction: well done and clear, improve your introduction with aspect discussed in this article (Grabovac I, Veronese N, Stefanac S, et al. Human Immunodeficiency Virus Infection and Diverse Physical Health Outcomes: An Umbrella Review of Meta-analyses of Observational Studies. Clin Infect Dis. 2020;70(9):1809‐1815.)

Response: We agree with suggestion and appreciate the reference. We provided additional context by describing the role of the HIV continuum of care and reaching viral suppression. In lines 54-60 and 73-76.

4) Discussion:
Reviewer comment: improve your discussion with data on how low socioeconomic levels are risk factors for HIV and other infectious diseases (Di Gennaro F, et al. Prevalence and Predictors of Malaria in Human Immunodeficiency Virus-Infected Patients in Beira, Mozambique. Int J Environ Res Public Health. 2018;15(9):2032. Published 2018 Sep 17)

Response: We appreciate the reference and incorporated it into our revision found in lines 577-586

Reviewer comment: and how the sharing of knowledge is the base of task shifting and has a central role to change health in more vulnerable people. (Marotta C, et al. Pathways of care for HIV infected children in Beira, Mozambique: pre-post intervention study to assess the impact of task shifting. BMC Public Health. 2018;18(1):703. Published 2018 Jun 7.)

Response: While task shifting is a potentially effective approach to address the human resource limitations and to scale up the ART coverage and retention in care, we could not find any evidence of the role of task shifting to address specific barriers and facilitators faced by Latinos in the US. However, we did identify evidence of the impact that culturally appropriate HIV care can have on retention in care, and delivering care via telemedicine to reach Latinos in areas where PrEP and HIV care may not be readily available. This is described in lines 507-523.

We appreciate the reviewer’s comments and insights that guided our development of a more organized and comprehensive manuscript. Thank you for your consideration of this article for publication in PLOS ONE.

Sincerely,

Nanette Benbow M.A.S.

Center for Prevention Intervention Methodology (Ce-PIM)

Northwestern University
