# Supplementary material for: County-level factors affecting Latino HIV disparities in the United States
Source: PLoS One. 2020 Aug 12;15(8):e0237269. doi: 10.1371/journal.pone.0237269 (PMC7423131; doi:10.1371/journal.pone.0237269)
Supplement: Attachment — Submitted filename: Reviewer Comments.docx [file pone.0237269.s003.docx]

The manuscript is interesting and could be accepted after revision.

Authors should improve the discussion, better explaining the lesson learnt and possible solutions to this problem.

Specifically:

1) Authors stated: “Increasing the availability, initiation, and retention of PrEP and ART are essential strategies to eliminate HIV.”….It has to be better contextualised and explained. In which way? In fact, in the previous paragraph they stated “In such settings, language and cultural differences could be significant barriers to HIV testing and treatment that would differentially affect Latinos across both HIV prevention and treatment cascades”…How authors think possible increase PreP and ART? See ref: DOI: 10.1186/s12889-018-5646-8

2) Authors should better explain also the following: “We conjecture that focusing primarily on changing individual level risk behavior by itself is not likely to overcome all these disparities” how much important it this for authors?

3) Authors should create a priorities list of actions to tackle this problem.

4) social context was mentioned many time but never well discussed, maybe authors could consider these references: https://doi.org/10.1016/j.tube.2017.01.002; doi: 10.1177/00333549101250S405
